# Delayed elimination communication is a crucial factor in disposable diaper dependence in Chinese preschool-aged children

**DOI:** 10.3389/fped.2022.1053118

**Published:** 2023-01-09

**Authors:** Jia-ting Yu, Qiu-fang Mao, Feng-ping Ji, Ying Zhao, Hui-jie Hu, Yan-ping Zhang, Jing Yang, Qing-wei Wang, Wei Lu, Jian Guo Wen

**Affiliations:** ^1^ Henan Joint International Pediatric Urodynamic Laboratory, The First Affiliated Hospital of Zhengzhou University, Zhengzhou, China; ^2^ Department of Urology, Third Affiliated Hospital of Xinxiang Medical University, Xinxiang, China; ^3^ Department of Urology, Nursing School Affiliated Hospital of Xinxiang Medical University, Xinxiang, China; ^4^ Department of Humanities Nursing, Sanquan College, Xinxiang Medical University, Xinxiang, China; ^5^ Department of Urology, Affiliated Xinyang Hospital, Zhengzhou University & Xinyang Central Hospital, Xinyang, China

**Keywords:** disposable diaper dependence, elimination communication, nomogram, factors, children

## Abstract

**Purpose:**

Elimination communication (EC) is considered to be a milestone in a child's development. Nowadays, a trend toward an older age at EC initiation has been observed globally, probably due to the convenience of disposable diaper use in daily life. The study aimed to identify potential risk factors for disposable diaper dependence (DDD) and evaluate whether an early/proper EC can reduce the risk of DDD among children in China.

**Methods:**

A cross-sectional study was performed on 13,500 children in mainland China from October 2019 to March 2020. An anonymous questionnaire was used to collect information including the sociodemographic characteristics, details about DDD and EC, and the effect of DDD on the quality of life of children. Data were analyzed by SPSS and R software.

**Results:**

The overall prevalence of DDD was 4.17% (4.31% in boys; 4.02% in girls) and decreased with age, from 8.71% at 2 years to 0.73% at 6 years (*χ*^2^*_trend_* = 210.392, *P* < 0.001). In univariable analysis, age, location or EC were associated with DDD. Four independent factors—age, location (urban), caregivers with high education levels (junior college or above) and delayed EC (after 12 months of age)—were identified to be significantly associated with DDD risk in logistic regression model. Compared with EC onset after 12 months of age, EC onset before 12 months of age was associated with a 79.6% (model 2) reduction in DDD. Four independent factors were selected to establish the nomogram for DDD based on the results of logistic regression analysis. The C-index (0.770) and the AUC (>0.7) indicated satisfactory discriminative ability of the nomogram. The calibration diagrams showed favorable consistency between the prediction of the nomogram and actual observations.

**Conclusion:**

Our findings indicate the joint contribution of age, location, caregivers’ education level and EC to DDD in Chinese preschool-aged children. Timely cessation of the use of disposable diapers and early/proper EC may help to reduce the risk of DDD in children.

## Introduction

In recent decades, the development of disposable diapers (DDs) has changed child care. DDs are comfortable, superabsorbent, and easy to use and dispose of in contrast with cloth diapers (1, 2). Consequently, a trend toward an older age at elimination communication (EC) initiation has been observed, as the convenience of DDs has likely led some parents to delay EC (3–5).

EC, an important milestone in a child's development, refers to a parenting practice that involves teaching infants toilet signals and schedules and physically assisting infants during urination and defecation starting in early infancy. Conversely, with toilet training, parents teach the child to toilet themselves and it typically starts at an older age compared to EC (4, 6). When the infant shows cues of elimination (crying, squirming, straining, wriggling, grimacing, fussing or vocalizing), the caregiver can coordinate this elimination process with audio cues (soft whistle or hum) whilst cradling the infant gently and non-coercively in a supported, secure squatting position to eliminate (4).

With rapid economic growth and information expansion, the use of DDs has been rapidly popularized and has impacted the traditional concept of parenting in China. We see an increasing number of parents with young children who heavily rely on DDs while ignoring the practice of EC daily in our clinical practice. Phenomena like reliance on diapers, refusal to eliminate, and crying and other symptoms at the time of DD removal were called disposable diaper dependence (DDD); we speculate DDD can be closely related to delayed EC.

In a previous study, we found that prolonged use of DDs and a later onset of EC in infants contributed to increases in the prevalence rates of primary nocturnal enuresis in children over 6 years of age and daytime urinary incontinence (DUI) in children aged 3–10 years in mainland China during the past 10 years (7, 8). However, the prevalence of DDD and its risk factors in children are still unknown. Moreover, the optimal age for the initiation of EC is controversial in the literature (1–4) and varies widely among different generations, countries and sociocultural groups (2, 4, 9).

In this cross-sectional study, we aimed to investigate the practice of EC and the age at daytime DD use cessation in children aged 2–6 years to identify potential risk factors for DDD and evaluate whether an early/proper EC can reduce the risk of childhood DDD.

## Participants and methods

### Study participants and exclusion criteria

**Figure 1:**
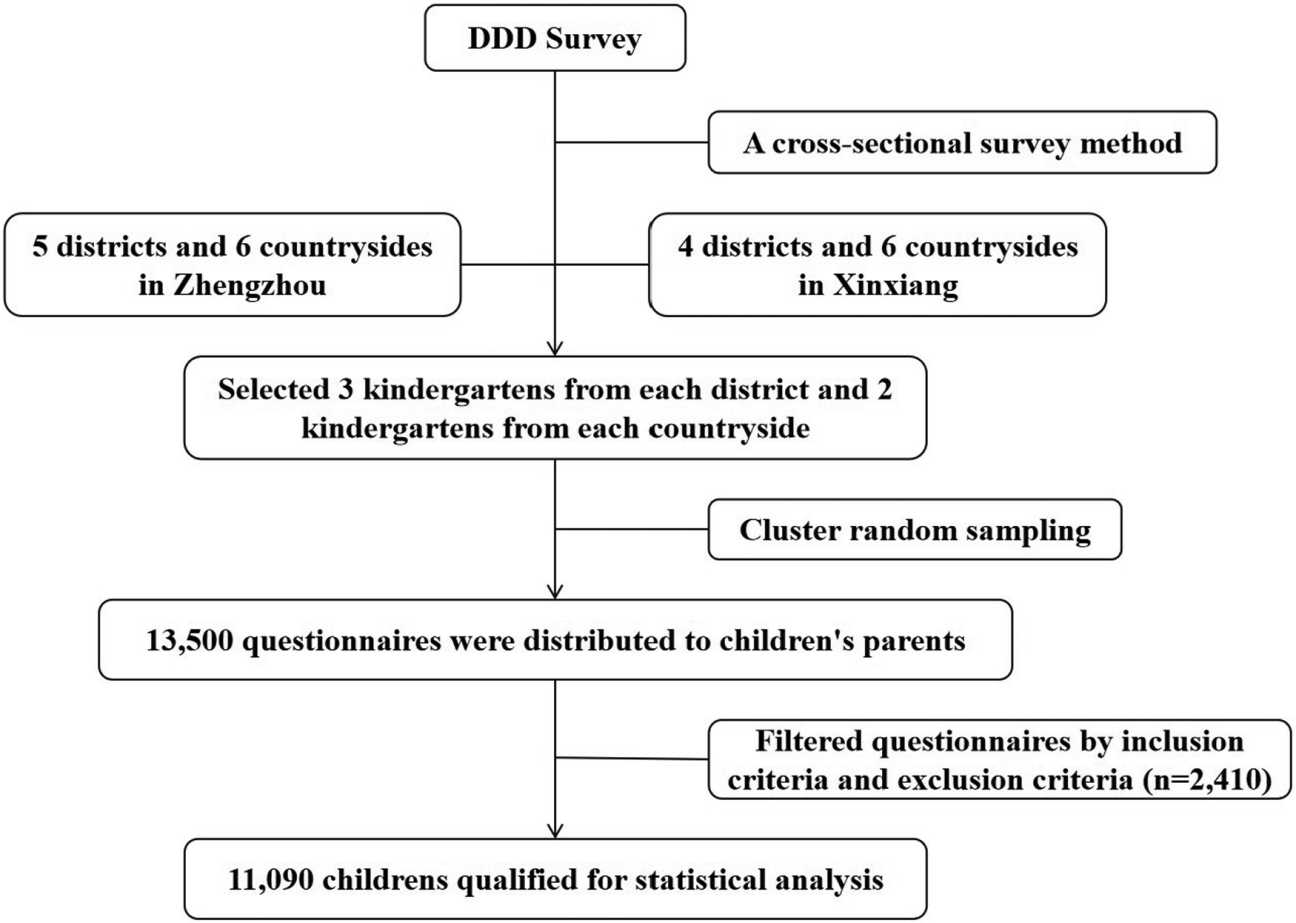
Disposable diaper dependence (DDD) survey flow chart.

From October 2019 to March 2020, an epidemiological survey of DDD among children aged 2–6 years was carried out in Zhengzhou city and Xinxiang city in Henan Province, China, by using multistage, stratified cluster random sampling and cross-sectional survey methods (Figure 1). Three kindergartens in each district and two kindergartens in each countryside were randomly selected for this study. Cluster sampling was used to select eligible kindergarten classes by using class as the unit of randomization. The questionnaire was distributed to the caregivers of 13,500 children, who were eligible for the study.

The inclusion criteria were healthy children without disabilities who were aged 2–6 years, had used diapers after birth and did or did not currently use diapers; informed consent obtained from a parent or guardian. The contents of and information about the study were fully explained to subjects. The exclusion criteria were functional or anatomical abnormalities in the urinary tract, nervous system or gastrointestinal tract; any other disorder affecting the lower urinary tract or bowel function; or incomplete or unreasonable answers on the questionnaire.

The definition of DDD was as follows: DD use since birth and heavy dependence on DDs after the age of 2 years; crying, resisting and refusing to cooperate with parental instructions, an inability to control urination and/or defecation (gatism) when a DD is not applied; and a restored calm state after DD application.

### Content of the questionnaire

A self-administered internet or in-person anonymous questionnaire was used to collect information. With the permission and cooperation of teachers of the surveyed kindergarten or parents of nursery school students, the questionnaire were distributed in parent meetings. The survey was carried out by trained investigators who explained the purpose and content of the survey to guardians and teachers. The significance of this survey and its positive effect on children's growth and development were briefly described, and free medical consultation was offered. A parent or guardian provided informed consent during the process of survey completion.

The questionnaire consisted of three parts. Part 1 investigated the demographic characteristics of the child and family, such as age (date of birth), sex, place of residence (urban or rural), primary caregiver and the primary caregiver's education level and occupation. Part 2 consisted of questions about the details of the use of DDs and EC, including whether DDs were used after birth; average daily usage; daytime DD use cessation age; DDD signs after DD use cessation; whether EC was practiced; onset time and frequency of EC; attitude toward EC; etc. The last part asked whether DDD affected the quality of life of the children. The response to the question, “How much does incontinence affect your child's daily life?”, was used to assess the effect of DDD on quality of life. The scale measures the effect of long-term use of diapers on daily life and school performance. The scale ranges from 0 to 3, with increments of 1, where no effect is scored as 0, little effect is scored as 1, moderate effect is scored as 2, and major effect is scored as 3. In addition, the last part contained questions about the occurrence of diaper-related adverse reactions, such as diaper rash and recurrent urinary tract infection (rUTI).

### Statistical analysis

Questionnaire data were double entered and validated using EpiData 3.1 software (The EpiData Association, Odense, Denmark). Statistical analyses were performed using IBM SPSS Statistics version 21.0 software (IBM, Armonk, NY, USA) and R 4.2.0 (Daniel E. Ho, Stanford, CA, USA). GraphPad Prism 8.0 (GraphPad Software Inc., San Diego, CA, USA) was used for graphing.

Continuous variables with skewed distributions are described as medians (25% percentile, 75% percentile) and were compared using the Mann‒Whitney *U* test. Categorical variables are presented as frequencies (percentages) and were analyzed with the Pearson chi-square test or chi-square test for trends. Pairwise comparisons were made with Bonferroni correction, and the significance threshold was adjusted to 0.008 (0.05/6). For the binary logistic regression model, candidate variables (confounders) with a *P* value <0.10 in the univariate analyses were included, and stepwise regression was performed. Then, based on the independent risk factors, a nomogram was established using the RMS package in R software.

The nomogram has been accepted as a reliable tool that quantifies the risk of a clinical event (10, 11). The discrimination and calibration of the nomogram were evaluated by the concordance index (C-index), area under the receiver operating characteristic (ROC) curve (AUC), and calibration diagrams. Decision curve analysis (DCA) was used to evaluate the clinical usefulness of the nomogram. The level of statistical significance was set to 0.05.

## Results

### Overall prevalence of DDD

**Figure 2:**
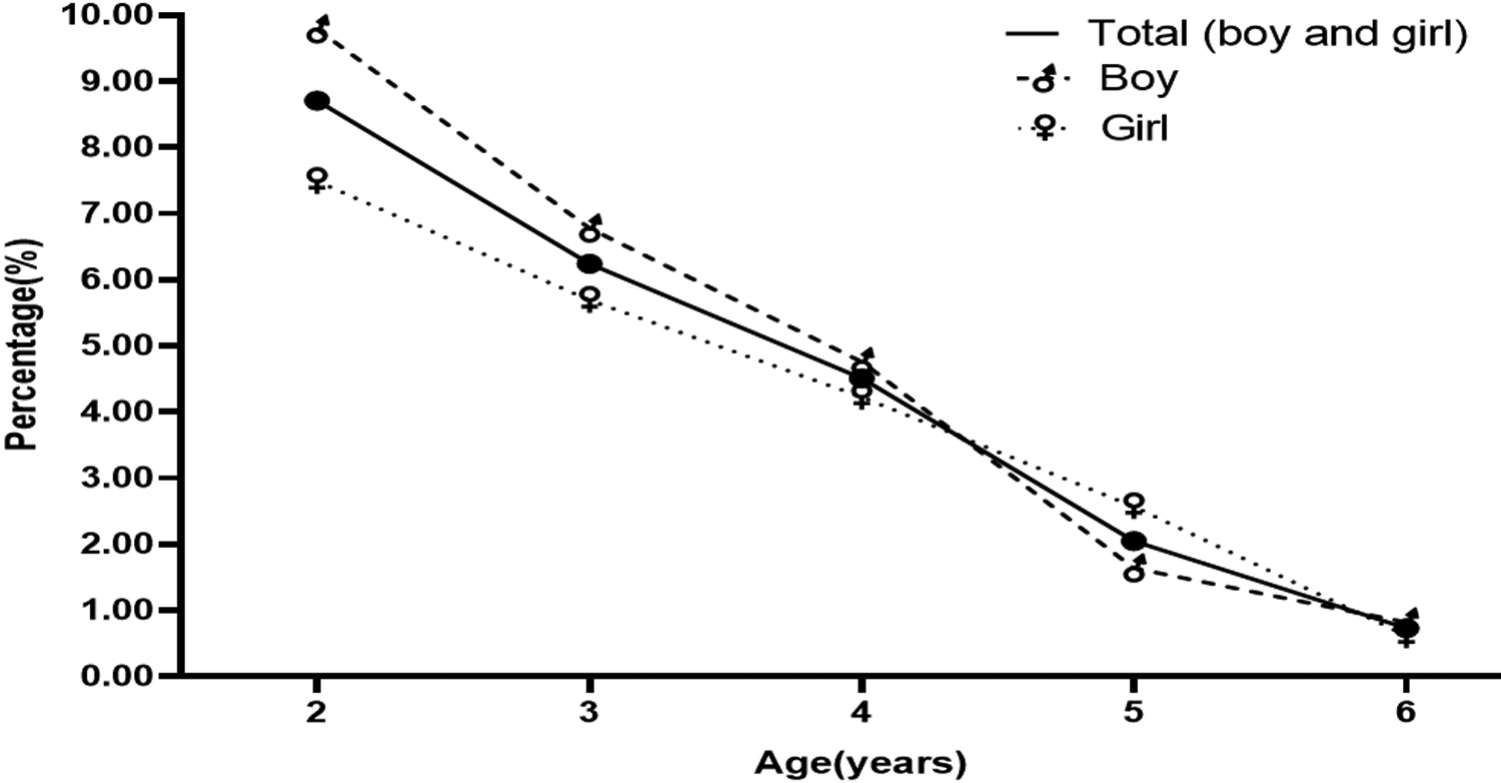
Proportion and prevalence of disposable diaper dependence (DDD) in children aged 2–6 years.

We distributed 13,500 questionnaires, and participants returned 11,090 (82.15%) valid questionnaires. More than half (5,985, 53.97%) of the participants were boys. Table 1 lists the distributions of participants’ characteristics among the study children. The overall prevalence of DDD was 4.17%, and the prevalence was 4.31% in boys and 4.02% in girls. No significant difference was found between boys and girls in any age group. With increasing age, the prevalence of DDD declined from 8.71% at 2 years to 0.73% at 6 years (*χ*^2^*_trend_*  = 210.392, *P* < 0.001), and the frequency of DDD in boys and girls both showed downward trends (Figure 2).

**Table 1:** Sociodemographic and clinical characteristics of the study patients (*n* = 11,090) by disposable diaper dependence (DDD).

Variables	Total (*n* = 11,090)	DDD (*n* = 463)	Non-DDD (*n* = 10,627)	*P*-value
Age at baseline, years	4	3 (2, 4)	4 (3, 5)	<0.001
2	1,838	160 (34.56%)	1,678 (15.79%)	<0.001*
3	2,051	128 (27.65%)	1,923 (18.10%)	
4	2,284	103 (22.25%)	2,181 (20.52%)	
5	2,727	56 (12.10%)	2,671 (25.13%)	
6	2,190	16 (3.46%)	2,174 (20.46%)	
Gender				0.439
Boy	5,985	258 (55.72%)	5,727 (53.89%)	
Girl	5,105	205 (44.28%)	4,900 (46.11%)	
Location				<0.001
Urban	5,724	281 (60.69%)	5,443 (51.22%)	
Rural	5,366	182 (39.31%)	5,184 (48.78%)	
Primary caregiver				0.067
Parents	6,411	268 (57.88%)	6,143 (57.81%)	
Grandparents	4,366	174 (37.58%)	4,192 (39.45%)	
Others	313	21 (4.54%)	292 (2.75%)	
Caregivers’ education level				0.066
High school diploma or less	5,206	198 (42.76%)	5,008 (47.13%)	
Junior college or above	5,884	265 (57.24%)	5,619 (52.87%)	
Caregivers’ occupation				0.754
Workers	2,669	112 (24.19%)	2,557 (24.06%)	
Farmers	2,443	95 (20.52%)	2,348 (22.09%)	
Administrator	1,628	74 (15.98%)	1,554 (14.62%)	
Businessman	1,854	81 (17.49%)	1,773 (16.68%)	
Kindergarten teacher	170	4 (0.86%)	166 (1.56%)	
Others	2,326	97 (20.95%)	2,229 (20.97%)	
Attitude to EC				0.084
Positive	5,272	198 (42.76%)	5,074 (47.75%)	
Negative	2,034	98 (21.17%)	1,936 (18.22%)	
Unclear	3,784	167 (36.07%)	3,617 (34.04%)	
EC				<0.001
Yes	9,856	347 (74.95%)	9,509 (89.48%)	
No	1,234	116 (25.05%)	1,118 (10.52%)	
EC onset time				<0.001*
≤12 months of age	7,471	177 (38.23%)	7,294 (68.64%)	
13–24 months of age	1,872	130 (28.08%)**	1,742 (16.39%)	
>24 months of age	513	40 (8.64%)**	473 (4.45%)	
Never training	1,234	116 (25.05%)**	1,118 (10.52%)	

* *P*-value for Chi-squared test for trend, and other *P*-value for Chi-squared test or Mann–Whitney *U* test.

** Compared with ≤12 months of age, *P* < 0.008 (Bonferroni correction).

### Risk factors of DDD as results of univariate analyses

The univariate analyses showed no significant difference in the prevalence of DDD by gender, primary caregiver, attitude to EC, caregivers’ education level or occupation. The prevalence rates of DDD in those living in urban areas and in those who never practiced EC were higher than that in those living in rural areas and in those who practiced EC, respectively (*P* < 0.001). Among the 11,090 children, 9,856 (88.87%) children practiced EC. Stratification by EC onset time showed that the incidence of DDD gradually increased with a later onset of EC (*χ*^2^*_trend_*  = 179.607, *P* < 0.001). *Post hoc* two-by-two paired comparison using the Bonferroni method to correct the significance level showed significant differences in the incidence of DDD among children aged 13–24 months, more than 24 months and less than 12 months who practiced EC.

### Risk factors of DDD as results of logistic regression analyses

To control for confounding effects on EC while analyzing the relationship between DDD and EC, a logistic regression model was used for analysis, with the results presented in Table 2. Three models were constructed: the unadjusted model; model 1, which controlled for age and location; and model 2, which was the same as model 1 and additionally controlled for primary caregiver, caregiver education level and attitude toward EC.

**Table 2:** Logistic regression models for disposable diaper dependence (DDD) (*n* = 11,090).

	Unadjusted Model		Model 1		Model 2	
	OR (95% CI)	*P*-value	OR (95% CI)	*P*-value	OR (95% CI)	*P*-value
EC onset time (reference: Never training)						
≤12 months of age	0.234 (0.184–0.298)	<0.001	0.206 (0.161–0.264)	<0.001	0.204 (0.158–0.263)	<0.001
13–24 months of age	0.719 (0.554–0.934)	0.013	0.755 (0.577–0.989)	0.041	0.767 (0.582–1.012)	0.060
>24 months of age	0.815 (0.560–1.186)	0.285	1.116 (0.757–1.645)	0.581	1.124 (0.759–1.665)	0.560
Age	NA	NA	0.561 (0.519–0.606)	<0.001	0.563 (0.521–0.608)	<0.001
Location (reference: Rural)						
Urban	NA	NA	1.401 (1.150–1.706)	0.001	1.353 (1.107–1.655)	0.003
Primary caregiver (reference: Others)						
Parents	NA	NA	NA	NA	0.904 (0.545–1.497)	0.694
Grandparents	NA	NA	NA	NA	0.918 (0.559–1.509)	0.737
Caregivers’ education level (reference: Junior college or above)						
High school diploma or less	NA	NA	NA	NA	0.748 (0.590–0.950)	0.017
Attitude to EC (reference: Unclear)						
Positive	NA	NA	NA	NA	0.917 (0.737–1.141)	0.436
Negative	NA	NA	NA	NA	1.058 (0.810–1.381)	0.678

Model 1 was adjusted for age (continuous variable), location; Model 2 was adjusted for the variables listed in the Model 1 as well as primary caregiver, caregivers’ education level and attitude to EC.

The results showed that the adjusted odds ratios (ORs) for DDD were 0.206 [95% confidence interval (CI), 0.161–0.264, model 1] and 0.204 (95% CI, 0.158–0.263, model 2) in individuals with an EC onset time before 12 months of age depending on the model used; additional sociocultural factors were accounted for in model 2. EC failure was a risk factor for DDD in children (*P* < 0.001). Compared with an EC onset time at more than 12 months of age, starting EC before 12 months of age was associated with a 79.6% (model 2) reduction in DDD. EC started before 12 months of age appeared to be a protective factor against DDD. In addition, the multivariate regression analysis also identified age (OR, 0.563; 95% CI, 0.521–0.608), location (OR, 1.353; 95% CI, 1.107–1.655), and caregivers’ education level (OR, 0.748; 95% CI, 0.590–0.950) as independent predictors of DDD.

### Diagnostic nomogram construction and validation

**Figure 3:**
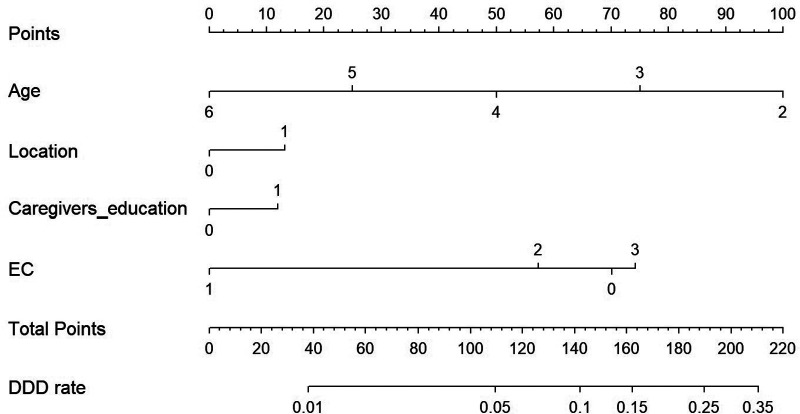
A constructed nomogram for diagnostic prediction of a patient with disposable diaper dependence (DDD). Draw a line perpendicular from the corresponding axis of each variable until it reaches the top line labeled “Points”. The sum of these numbers is located on the Total Points axis, and a line is drawn downwards to the DDD rate axis to identify the likelihood of DDD. For location, 0 = Rural and 1 = Urban. For education, 0 = High school diploma or less, and 1 = Junior college or above. For EC, 0 = Never training, 1 = ≤12 months of age, 2 = 13–24 months of age, and 3 = >24 months of age.

**Figure 4:**
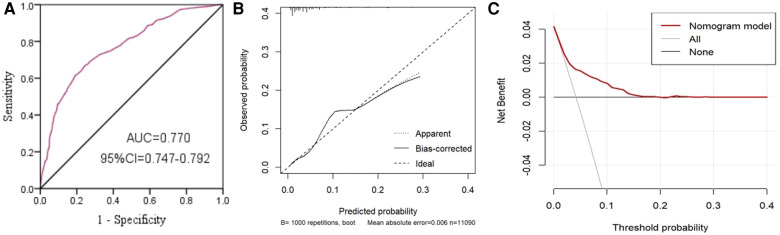
Area under the ROC curve (AUC) (**A**), calibration curve (**B**) and decision curve analysis (DCA) curve (**C**) of the nomogram.

We constructed the risk assessment nomogram according to the variables screened in model 2, including age, location, caregivers’ education level and EC (Figure 3). The C-index of the nomogram was 0.770, showing good discrimination ability. The calibration curve showed high consistency between the predicted and observed results. In addition, the DCA curve showed that the nomogram had net benefits and excellent performance in clinical practice (Figure 4).

### Implications of DDD in the overall quality of life

The effect of DDD of the children's quality of life was analyzed in the last domain of the questionnaire. The incidence of frequency and urgency in children with DDD was higher than that in normal children [39.31% (182/463) vs. 18.22% (1,936/10,627), *P* < 0.001], and 29 children (6.26%) with DDD had rUTIs (Table 3). The incidence of DD-related dermatitis in children with DDD was also higher than that in children without DDD [48.16% (223/463) vs. 29.52% (3,137/10,627), *P* < 0.001] (Table 3). Among the children with DDD, approximately 37% of parents or caregivers believed that DDD was not favorable to the child's overall daily life and normal school attendance, with varying negative impacts on quality of life.

**Table 3:** Adverse effects associated with disposable diaper dependence (DDD) (*n* = 11,090).

	DDD (*n* = 463)	Non-DDD (*n* = 10,627)	*P*-value
Frequency/Urgency	182 (39.31%)	1,936 (18.22%)	<0.001
rUTI	29 (6.26%)	217 (2.04%)	<0.001
DD-related dermatitis	223 (48.16%)	3,137 (29.52%)	<0.001

## Discussion

Diapering has been a global practice for centuries, as newborns are not capable of controlling urination and defecation after birth, and various methods and materials, such as cloth, are used depending on the tradition, culture, and geography of the people involved (1). The development of the DD occurred in the 20th century to meet the changing needs of people, and DDs now universally occupy the majority of the market due to their superabsorptive properties, increased comfort, antibacterial properties, and ease of use and disposal compared with cloth diapers; many countries have discontinued the use of cloth diapers (1, 4).

However, cloth diapers have limited fluid absorption capacity, which facilitates the EC process, and children in cloth diapers tend to end toilet training sooner than those in modern DDs.

With the popularization of DD use (1, 12), the phenomenon of severe DDD has gradually received attention. The prevalence of DDD in the current study was 8.71% in children aged 2 years and decreased with age to 4.51% in those aged 4 years. The kindergarten admission age is 2–3 years old; such children are still highly dependent on diapers, which not only increases the workload of kindergarten teachers but also puts children in stressful situations due to embarrassment and can have a negative psychological impact on them. In addition, knowledge of bladder function development is not universal, and many children with DDD are frequently considered to have urinary incontinence by parents and kindergarten teachers. Therefore, it is crucial to survey the prevalence of DDD and identify the risk factors for DDD in children and to initiate early preventive interventions in children.

There are differences in the toilet training onset age among countries and cultures. Globally, attitudes and practices toward EC have changed constantly for decades, and children are initiating and completing EC later than in previous generations (2, 4, 9, 13, 14). The age of initiation of EC has progressively increased in the USA from under 18 months in the late 1940s to 21-36 months today (12, 13). Caucasian parents believed that EC should be initiated at a later age (25.4 months) than that believed by African American (18.2 months) or other parents (19.4 months) (9). Mean initiation of EC age was 22.05 months in Turkey (15). Duong et al. reported that the vast majority of Vietnamese children initiated EC at the age of 6 months, while only a few Swedish children had initiated EC by the age of 24 months (16). Most countries in Asia and Africa generally start training earlier than Western countries (4, 15–20). The American Academy of Pediatrics (AAP) recommends that EC training should begin at age 18–24 months or later but also noted that initiating EC before 18 months is unlikely to do any damage (15, 21). In China, many parents of infants traditionally practice “Baniao” (a Chinese term), described as lifting of the child in a semisquatting position with their thighs apart over the toilet or potty, similar to EC (22). Recent studies have shown that neural pathways related to bladder control exist after birth, and neonatal sleeping electroencephalogram (EEG) recordings show that bladder filling induces marked increases in cortical discharge, indicating that newborns and infants have advanced central involvement in urination (23, 24). Some investigators have demonstrated that earlier EC is likely to lead to early control during the day and at night without bladder dysfunction (18, 25–28). In this study, we found that a higher DDD risk was associated with a later onset time of EC, as well as a longer usage time of DDs. The advantage of early EC to prevent DDD was independent of potential confounders (sex, caregiver's work status, etc.). Compared with an EC onset time at more than 12 months of age, starting EC before 12 months of age was associated with a 79.6% (model 2) reduction in DDD. This study suggests that early training may decrease the incidence of DDD. It is acceptable to urinate in public in rural areas in China, which makes it easier for children to remain dry. Urban families of children, due to the concept of Western parenting and marketing promotions, use DDs more frequently, and urban regions limit EC. Beginning EC at a younger age would reduce the increasing number of DDs used, which has environmental advantages (25, 26). The Western regimen is probably not feasible in Chinese society, where children usually go to kindergarten at the age of 2–3, as kindergarten teachers need to assist children who have difficulty in urination and defecation, which markedly increases the burden on kindergarten teachers. On the other hand, delayed EC could psychosocially affect the individual as well as their family and cause contemporary problems, such as school bullying and discrimination (29–31).

Furthermore, the overuse of DDs has adverse effects on children's quality of life and growth and development. rUTI is one of the most common bacterial infections in children and can involve the kidneys, leading to renal scarring and related long-term complications, such as chronic kidney disease and hypertension (32). Studies have shown that long-term use of DDs in children increases the risk of UTI (19, 20), which could be due to maturational delay or behavioral issues leading to either infrequent voiding or incomplete voiding which are sources of stasis and the primary cause of UTI. The results of this study showed that 39.31% of the children with DDD had symptoms of UTI, and 6.26% of them had rUTIs. Duong et al. advocated that infants should practice early EC, which can promote bladder emptying and reduce the incidence of UTI (33).

In addition, diaper-related dermatitis is a common complication in children who use DDs and manifests as an acute exacerbation of inflammation (34). The incidence of DD-associated dermatitis was 48.16%. The incidence rates of these conditions were significantly higher than those in the non-DDD group.

DDs are also associated with environmental concerns, such as water, air, and soil pollution, resource consumption, and waste production. Some studies have estimated that more than 22 kg of petroleum and 136 kg of wood are needed to produce DDs for one baby per year (35, 36). Because of their relatively high price, biodegradable single-use diapers are the choice of few families (37). Hence, the rational use of DDs and timely EC are crucial for the prevention of DDD in children.

In our study, we also created a simple and intuitive predictive nomogram that quantifies the risk of DDD. We identified four factors—age, location, caregivers’ education level and EC—were predictive of DDD, and our nomogram predicts patient-specific probabilities of DDD with optimal discrimination and excellent calibration.

As a cross-sectional analysis, the current study is not devoid of limitations. One of the limitations of this study is that biases could not be avoided completely during the collection of data. We were unable to include other factors that could have influenced our study such as psychiatric, intellectual and behavioral problems in either the parents or the child, and diseases with inherited traits, which will be investigated in future research. Multicenter prospective cohort studies are needed to verify our results and to further clarify the role of EC in the prevention of DDD and its impact on children's physical and mental development.

## Conclusion

In summary, DDD is a phenomenon characterized by delayed physiological development of urination and defecation control possibly caused by the overuse of DDs and lack of EC. This study indicated that DDD is determined by the joint contribution of multiple potential factors including age, location, caregivers’ education level, and EC in Chinese preschool-aged children. It is suggested that the appropriate time to start EC is before the age of 12 months, and DD use should be terminated according to the child's urination and defecation control ability. The diagnostic nomogram constructed and validated in this study can be used as an auxiliary tool to identify DDD in children and assist clinicians in making more scientific clinical decisions.

## Data Availability

The raw data supporting the conclusions of this article will be made available by the authors, without undue reservation.
